# Outcome reporting bias in trials: a methodological approach for assessment and adjustment in systematic reviews

**DOI:** 10.1136/bmj.k3802

**Published:** 2018-09-28

**Authors:** Jamie J Kirkham, Douglas G Altman, An-Wen Chan, Carrol Gamble, Kerry M Dwan, Paula R Williamson

**Affiliations:** 1 MRC North West Hub for Trials Methodology Research, Department of Biostatistics, University of Liverpool, Liverpool L69 3GL, UK; 2 Centre for Statistics in Medicine, Nuffield Department of Orthopaedics, Rheumatology and Musculoskeletal Sciences, University of Oxford, Oxford, UK; 3 Department of Medicine, Women’s College Research Institute, Women’s College Hospital, University of Toronto, Toronto, ON, Canada; 4 Cochrane Editorial Unit, London, UK

## Abstract

Systematic reviews of clinical trials aim to include all relevant studies conducted on a particular topic and to provide an unbiased summary of their results, producing the best evidence about the benefits and harms of medical treatments. Relevant studies, however, may not provide the results for all measured outcomes or may selectively report only some of the analyses undertaken, leading to unnecessary waste in the production and reporting of research, and potentially biasing the conclusions to systematic reviews. In this article, Kirkham and colleagues provide a methodological approach, with an example of how to identify missing outcome data and how to assess and adjust for outcome reporting bias in systematic reviews.

“Trials that presented findings that were not significant (P≥0.05) for the protocol-defined primary outcome in the internal documents either were not reported in full or were reported with a changed primary outcome.”^1^

Selective reporting of outcome data creates a missing data problem. Bias arises when trialists select outcome results for publication based on knowledge of the results. Hutton and Williamson first defined outcome reporting bias (sometimes termed selective reporting bias) in 2000: “the selection on the basis of the results of a subset of the original variables recorded for inclusion in a publication.”^2^

Empirical research provides strong evidence that outcomes that are statistically significant have higher odds of being fully reported than non-significant outcomes (odds ratios ranging from 2.2 to 4.7).^3^ ^4^ In the ORBIT (Outcome Reporting Bias In Trials) study, outcome reporting bias was suspected in at least one trial in more than a third (96/283; 34%) of Cochrane systematic reviews.^5^ In the follow-up study that looked at the same problem in a review of harm outcomes, review primary harm outcome data were missing from at least one eligible study in over 75% (252/322)****of systematic reviews.^6^

The aim of this article is to show, with an example, how systematic reviewers can minimise the amount of missing data in reviews of healthcare interventions, and use ORBIT methods to detect and classify the suspicion of outcome reporting bias in benefits and harms reported in included studies. The paper also provides details of a statistical approach to assess the robustness of meta-analysis conclusions on this potential source of bias that non-methodologists can implement on a web based platform.

**Summary points**

- Outcome reporting bias occurs when trialists select for publication a subset of the original recorded outcomes based on knowledge of the results. Outcome reporting bias is a threat to evidence based medicine and contributes to waste in research
- Empirical evidence suggests that statistically significant outcomes have higher odds of being fully reported than non-significant outcomes (odds ratios ranging from 2.2 to 4.7)
- The ORBIT (Outcome Reporting Bias in Trials) research programme offers tools for systematic reviewers to identify missing outcome data, and assess and adjust for outcome reporting bias. A tutorial is provided to show how these tools could be used in a systematic review

## Selecting the most appropriate review outcomes

One way to streamline the process of systematic reviews and to help reduce outcome reporting bias is for reviewers to use outcomes considered as core for all trials in a particular topic area.^7^ These core outcome sets will ensure that every included trial can contribute data on key outcomes to the review analyses. An example is a set of core outcomes that have been developed for different skin conditions, which has now been endorsed by the Cochrane Skin Group Core Outcome Set Initiative (CSG-COUSIN).^8^

We recommend that systematic reviewers consider core outcome sets when registering their topics with Cochrane review groups or PROSPERO (international prospective register of systematic reviews). Systematic reviewers can identify if relevant core outcome sets exist for their review area by searching the publically accessible COMET (Core Outcome Measures in Effectiveness Trials) database.^9^

## Inclusion and exclusion criteria

Reviewers should not exclude studies from their review on the basis of whether the outcomes of interest were reported, because non-reporting does not necessarily mean that the outcomes were not measured.^5^ This recommendation also forms part of the mandatory methodological standards (item C40) for the conduct of new Cochrane intervention reviews (Methodological Expectations of Cochrane Intervention Reviews).^10^ Despite these recommendations and recent screening initiatives introduced by the Cochrane Editorial Unit, a quarter of Cochrane reviews are still excluding studies because of missing relevant outcome data.^11^

We strongly encourage systematic reviewers to include relevant studies in their review irrespective of whether the studies reported any of the review outcomes of interest, and to assess those studies in accordance with the methods described below.

## Identifying missing outcome data in reviews

**Fig 1:**
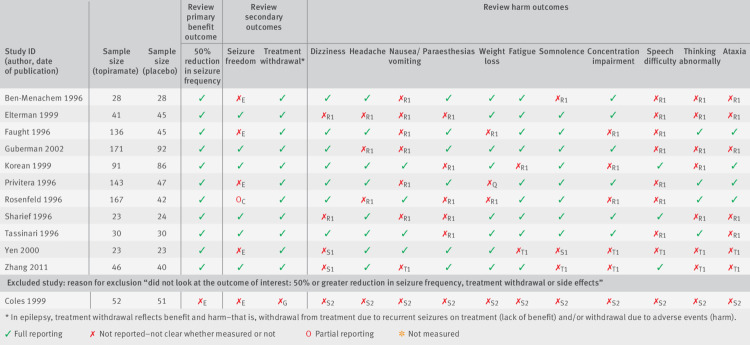
Outcome matrix for review on topiramate add-on use for drug resistant partial epilepsy, with letters indicating ORBIT (Outcome Reporting Bias In Trials) classifications

An outcome matrix was proposed to help identify missing study outcome data.^5^ Reviewers can construct the matrix with the outcomes of interest in the review, with those reported in the trial reports listed in the columns and the different studies listed in the rows. An example of an outcome matrix for a Cochrane systematic review, “Topiramate add-on for drug-resistant partial epilepsy,”^12^ is presented in figure 1. When this review was undertaken, there was no core outcome set that covered the scope of the review; therefore, the choice of outcomes was left to the discretion of the review authors. The aim of the review was to evaluate the efficacy and tolerability of topiramate when used as an add-on treatment for people with drug resistant partial epilepsy. The review considered two benefit and 12 harm outcomes. Eleven studies were included, while the review authors excluded one further study because there were “no relevant outcome data.” According to our earlier recommendation, the review authors should not have excluded this study (“Coles 1999”) from the review; we include this study in the example to provide a retrospective assessment of the impact of outcome reporting bias in this review. The outcome matrix, similar to the one presented in figure 1, provides a transparent way for systematic reviewers to show the outcomes that are reported for each study in the review, and those that are missing or partially reported. Partial reporting of outcome data refers to those that are inadequately reported for inclusion in a review meta-analysis (for example, an effect size was presented with no measure of precision or exact P value).

For trials that do not or partially report on outcomes, the matrix allows reviewers to assess the risk of outcome reporting bias according to the ORBIT classification system (see next section below). In the epilepsy example in figure 1, we can see from the matrix that all studies (apart from the excluded Cole 1999 study) reported data on 50% reduction in seizure frequency but only six of the studies fully reported data on seizure freedom. These two outcomes are structurally related; so if a trial author reports on one of these outcomes, this suggests that the trialist must have also measured the other outcome.

We strongly encourage systematic reviewers to include an outcome matrix in their reviews. The ORBIT matrix generator, available on the ORBIT website,^13^ is a tool that researchers can easily use to construct an ORBIT outcome matrix, which researchers can also export for inclusion in a systematic review article.

## Obtaining unpublished information

Sources for obtaining unpublished information on clinical trials for use in systematic reviews have previously been described.^14^ These sources (for example, trial registry and regulatory agency databases, trialist and sponsor contact, litigation documents, conference abstracts, and internet searches) can help identify unreported outcomes in published trials. To minimise the amount of missing data in reviews, we recommend that reviewers should attempt to obtain as much missing outcome data as possible. Reviewers can then update the outcome matrix to reflect information obtained from unpublished sources.

## Detecting outcome reporting bias

When no usable missing outcome data can be obtained from the sources given above, the ORBIT researchers developed classification systems to help systematic reviewers detect outcome reporting bias in reviews. The method relies only on information in the published trial report (for example, inconsistencies between sections such as the abstract, methods, and results), expert clinical judgment, and an assessment of the outcomes that researchers usually report across a set of trials in a review.^5^ The ORBIT researchers showed that the classification systems are able to detect bias with high sensitivity, even without access to other source documents to help perform the assessment, such as trial registry entries and study protocols.^5^ The classifications can also help reviewers judge the level of risk of bias within the “selective reporting outcome” domain by using the risk of bias tool currently used by the Cochrane Collaboration.^15^ Trials not reporting or partially reporting a review outcome should be labelled high risk, low risk, or no risk according to ORBIT terminology for benefit outcomes (table 1) or harm outcomes (table 2). The two classification systems identify whether: there is evidence that the trialist measured and analysed (or compared) the outcomes; the trialist measured but did not necessarily analyse (or compare) the outcomes; it is unclear whether the trialist measured the outcomes; or it is clear that the trialist did not measure the outcomes.

**Table 1:** The ORBIT (Outcome Reporting Bias In Trials) classification system for missing or incomplete outcome reporting in benefit outcomes. Figure 1 uses these classifications^5^

ORBIT classification	Description	Level of reporting	Risk of bias*
Clear that the outcome was measured and analysed			
A	Trial report states that outcome was analysed but only reports that result was not significant (typically stating P>0.05)	Partial	High risk
B	Trial report states that outcome was analysed but only reports that result was significant (typically stating P<0.05)	Partial	No risk
C	Trial report states that outcome was analysed but insufficient data were presented for the trial to be included in meta-analysis or to be considered to be fully tabulated	Partial	Low risk
D	Trial report states that outcome was analysed but no results reported	None	High risk
Clear that the outcome was measured			
E	Clear that the outcome was measured. Judgment says outcome likely to have been analysed but not reported because of non-significant results	None	High risk
F	Clear that the outcome was measured. Judgment says outcome unlikely to have been analysed	None	Low risk
Unclear whether the outcome was measured			
G	Not mentioned but clinical judgment says likely to have been measured and analysed but not reported on the basis of non-significant results	None	High risk
H	Not mentioned but clinical judgment says unlikely to have been measured at all	None	Low risk
Clear that the outcome was not measured			
I	Clear that the outcome was not measured	NA	No risk

NA=not appropriate.

* Risk of bias arising from the lack of inclusion of non-significant results when a trial was excluded from a meta-analysis or not fully reported in a review because the data were unavailable.

**Table 2:** The ORBIT II (Outcome Reporting Bias In Trials II) classification system for missing or incomplete outcome reporting in harm outcomes. Figure 1 uses these classifications^6^

ORBIT II classification	Description	Level of reporting	Risk of bias*
Explicit specific harm outcome: measured and compared across treatment groups			
P1	States outcome analysed but reported only that P>0.05	Partial	High risk
P2	States outcome analysed but reported only that P<0.05	Partial	High risk
P3	Insufficient reporting for meta-analysis or full tabulation	Partial	Low risk
Explicit specific harm outcome: measured but not compared across treatment groups			
Q	Clear that outcome was measured and clear outcome was not compared	NA	No risk
Explicit specific harm outcome: measured, not clear whether compared or not across treatment groups			
R1	Clear that outcome was measured but no results reported	None	High risk
R2	Result reported globally across all groups	None	High risk
R3	Result reported from some groups only	None	High risk
Specific harm outcome not explicitly mentioned: clinical judgment says likely measured and likely compared across treatment groups			
S1	Only pooled adverse events reported (could include specific harm outcome)	None	High risk
S2	No harms mentioned or reported	None	High risk
Specific harm outcome not explicitly mentioned: clinical judgment says likely measured but no events			
T1	Specific harm not mentioned but all other specific harms fully reported	None	Low risk
T2	No description of specific harms	None	Low risk
Specific harm outcome not explicitly mentioned, clinical judgment says unlikely measured			
U	No harms mentioned or reported	None	Low risk
Explicit the specific harm outcome was not measured			
V	Report clearly specifies that data on the specific harm of interest were not measured	NA	No risk

NA=not appropriate.

* Bias would occur if specific harm had been measured, but data were presented or suppressed in a way that would mask the harm profile of particular interventions.

For the epilepsy review, the classifications given for each outcome with missing or partially reported data are presented in the outcome matrix (fig 1), with a full justification of each classification listed in supplementary table 1, and if appropriate verbatim text taken from the trial report. These justifications can also be included in systematic reviews to support the risk of bias assessment. For example, in Cochrane reviews, authors are encouraged to provide “support for judgment” on each risk of bias judgment made.

To assess the risk of bias due to selective reporting, we recommend that the ORBIT classification system is used by systematic reviewers as a framework to determine whether there is a high or low risk of outcome reporting bias when review outcome data are missing or partially reported. We also recommend that the assessment is completed by at least two independent researchers and differences discussed to agree on an overall classification (in the epilepsy review, assessments were completed by two methodologists and a clinical neurologist).

## Adjusting for outcome reporting bias in systematic reviews

Copas and colleagues developed new statistical sensitivity approaches for assessing the robustness of review meta-analysis conclusions when outcome reporting bias is suspected.^16^ The Copas method takes into account the relative sample size of the studies with missing outcome data and directly models the mechanism of outcome reporting bias for benefit and harm outcomes. The Copas method is particularly attractive because it uses the high and low risk of bias classifications already assigned using the ORBIT system.

We return to our epilepsy example given earlier, for which the outcome matrix of the ORBIT classification is shown in figure 1. The Copas method can be implemented on a user friendly platform for fixed effects meta-analyses for binary data through the ORBIT website.^17^ Full instructions on how to set up the data frame and use this method in 10 simple steps are available on the website.^17^ The data frame for the epilepsy example can also be downloaded from the website. The method can be applied to other data types and random effects meta-analyses—the study team are currently working on implementing this on the same user friendly platform. Further support on using the Copas method and interpreting the data can be accessed through the “Contact” page of our website.^17^ ^18^

After applying the Copas bias adjustments for partially reported or unreported outcomes (table 3), the review had overestimated the benefits and underestimated the harms of the test treatment.^16^ The adjustment was greater for the harm outcomes, for which fewer studies reported the outcomes of interest, and less of a concern for the benefit outcomes, for which there were fewer missing outcomes. For example, when considering the harm outcome nausea and vomiting, the unadjusted estimate (relative risk 1.50, 95% confidence interval 0.71 to 3.15) reported in the review suggested no statistically significant difference between treatments. However, the Copas estimate after adjustment for outcome reporting bias (1.90, 1.08 to 3.59) suggested statistically significantly more harm in the treatment arm.

**Table 3:** Risk assessment of add-on use of topiramate for drug resistant partial epilepsy, accounting for partially reported and unreported study outcomes. Data are Mantel-Haenszel estimates and confidence intervals, unadjusted and adjusted for outcome reporting bias (using the Copas method)^16^

Outcome	Pooled estimate, relative risk (95% CI)	
	Unadjusted	Copas adjustment
Benefits		
50% seizure reduction	2.97 (2.38 to 3.72)	2.87 (2.31 to 3.57)
Seizure freedom	3.41 (1.37 to 8.51)	2.66 (1.19 to 5.78)
Harms		
Treatment withdrawal	2.44 (1.45 to 4.10)	2.47 (1.48 to 4.13)
Dizziness	1.54 (1.07 to 2.22)	1.64 (1.16 to 2.32)
Headache	0.99 (0.67 to 1.44)	1.14 (0.83 to 1.58)
Nausea and vomiting	1.50 (0.71 to 3.15)	1.90 (1.08 to 3.59)
Paraesthesias	3.91 (1.51 to 10.12)	4.40 (1.87 to 10.83)
Weight loss	3.47 (1.55 to 7.79)	3.60 (1.69 to 7.92)
Fatigue	2.19 (1.42 to 3.40)	2.22 (1.46 to 3.42)
Somnolence	2.29 (1.49 to 3.51)	2.35 (1.55 to 3.57)
Concentration impairment	7.81 (2.08 to 29.29)	8.25 (2.45 to 29.89)
Speech difficulty	3.37 (0.80 to 14.13)	4.48 (1.55 to 16.01)
Thinking abnormality	5.70 (2.26 to 14.38)	6.02 (2.54 to 14.79)
Ataxia	2.29 (1.10 to 4.77)	2.61 (1.36 to 5.16)

When outcome reporting bias is suspected, we recommend that the quality of evidence is lowered in relation to the standard GRADE (Grades of Recommendation, Assessment, Development and Evaluation) assessment applied in reviews. We also recommend that sensitivity analyses are performed for important outcomes to assess the robustness of conclusions to outcome reporting bias.^16^

## Conclusions

Empirical evidence suggests that outcome reporting bias is a threat to the validity of the evidence base and contributes to research waste. We have highlighted up-to-date approaches and recommendations for detecting this problem and adjusting the results when performing sensitivity analyses in systematic reviews. We anticipate continued application of these methods and methodological research into the assessment and adjustment of outcome reporting bias in the years to come. The ORBIT website is a useful resource, providing researchers with tools to detect outcome reporting bias and methods for sensitivity analysis; it is also a repository for important publications in this field.

## Web extra

Extra material supplied by authors

**Supplementary table 1:** ORB classification tables and justification

## Notes

Contributors: The authors include statisticians and a clinical epidemiologist with expertise in outcome reporting bias and a particular interest in methods and initiatives for reducing waste in research. All authors contributed to the planning, writing, and editing of this paper. JJK provided the illustrative example and is the guarantor. The corresponding author attests that all listed authors meet authorship criteria and that no others meeting the criteria have been omitted.
