# Supplementary material for: Outcome reporting bias in trials: a methodological approach for assessment and adjustment in systematic reviews
Source: BMJ. 2018 Sep 28;362:k3802. doi: 10.1136/bmj.k3802 (PMC6161807; doi:10.1136/bmj.k3802)
Supplement: Supplementary file 1 — Supplementary table 1: ORB classification tables and justification [file kirj043794.ww.pdf]

Supplementary Table 1: ORB classification tables with justification

Ben-Menachem 1996

Review outcome	Classification	Justification for classification
50% reduction in seizure frequency	NA	Outcome data reported in review meta-analysis.
Seizure freedom	E	50% reduction in seizure frequency was reported in this trial and therefore the trial must have measured seizure freedom. As the five studies reporting on seizure freedom reported non-significant results, it is likely that seizure freedom was analysed in this trial but not reported because of a non-significant result, especially as the 50% reduction in seizure frequency result was reported to be significant favouring topirimate.
Treatment withdrawal	NA	Outcome data reported in review meta-analysis.
Harms data were collected during patient interviews at each visit.		
Dizziness	NA	Outcome data reported in review meta-analysis.
Headache	NA	Outcome data reported in review meta-analysis.
Nausea/vomiting	R1	The AEs reported in the trials were only those that occurred in >=15% in either treatment group. Clear outcome was measured but not reported as unlikely to have met the reporting threshold.
Paraesthesias	NA	Outcome data reported in review meta-analysis.
Weight loss/decrease	NA	Outcome data reported in review meta-analysis.
Fatigue	NA	Outcome data reported in review meta-analysis.
Somnolence	R1	The AEs reported in the trials were only those that occurred in >=15% in either treatment group. Clear outcome was measured but not reported as unlikely to have met the reporting threshold.
Concentration impairment	NA	Outcome data reported in review meta-analysis.
Speech difficulty	R1	The AEs reported in the trials were only those that occurred in >=15% in either treatment group. Clear outcome was measured but not reported as unlikely to have met the reporting threshold.
Thinking abnormally	R1	The AEs reported in the trials were only those that occurred in >=15% in either treatment group. Clear outcome was measured but not reported as unlikely to have met the reporting threshold.
Ataxia	R1	The AEs reported in the trials were only those that occurred in >=15% in either treatment group. Clear outcome was measured but not reported as unlikely to have met the reporting threshold.

Etherman 1999

**Review outcome**	**Classification**	**Justification for classification**
50% reduction in seizure frequency	NA	Outcome data reported in review meta-analysis.
Seizure freedom	NA	Outcome data reported in review meta-analysis.
Treatment withdrawal	NA	Outcome data reported in review meta-analysis.
Harms data were collected by interviewing patients (or parents/guardians) in a non-directed manner.		
Dizziness	R1	The AEs reported in the trials were only those that occurred in >=10% in either treatment group. Clear outcome was measured but not reported as unlikely to have met the reporting threshold.
Headache	R1	The AEs reported in the trials were only those that occurred in >=10% in either treatment group. Clear outcome was measured but not reported as unlikely to have met the reporting threshold.
Nausea/vomiting	R1	The AEs reported in the trials were only those that occurred in >=10% in either treatment group. Clear outcome was measured but not reported as unlikely to have met the reporting threshold.
Paraesthesias	R1	The AEs reported in the trials were only those that occurred in >=10% in either treatment group. Clear outcome was measured but not reported as unlikely to have met the reporting threshold.
Weight loss/decrease	NA	Outcome data reported in review meta-analysis.
Fatigue	NA	Outcome data reported in review meta-analysis.
Somnolence	NA	Outcome data reported in review meta-analysis.
Concentration impairment	NA	Outcome data reported in review meta-analysis.
Speech difficulty	R1	The AEs reported in the trials were only those that occurred in >=10% in either treatment group. Clear outcome was measured but not reported as unlikely to have met the reporting threshold.
Thinking abnormally	R1	The AEs reported in the trials were only those that occurred in >=10% in either treatment group. Clear outcome was measured but not reported as unlikely to have met the reporting threshold.
Ataxia	R1	The AEs reported in the trials were only those that occurred in >=10% in either treatment group. Clear outcome was measured but not reported as unlikely to have met the reporting threshold.

Faught 1996

**Review outcome**	**Classification**	**Justification for classification**
50% reduction in seizure frequency	NA	Outcome data reported in review meta-analysis.
Seizure freedom	E	50% reduction in seizure frequency was reported in this trial and therefore the trial must have measured seizure freedom. As the five studies reporting on seizure freedom reported non-significant results, it is likely that seizure freedom was analysed in this trial but not reported because of a non-significant result, especially as the 50% reduction in seizure frequency result was reported to be significant favouring topirimate.
Treatment withdrawal	NA	Outcome data reported in review meta-analysis.
Harms data were recorded in the subject's diary and reviewed.		
Dizziness	NA	Outcome data reported in review meta-analysis.
Headache	NA	Outcome data reported in review meta-analysis.
Nausea/vomiting	R1	The AEs reported in the trials were only those that occurred in >=20% in either treatment group. Clear outcome was measured but not reported as unlikely to have met the reporting threshold.
Paraesthesias	NA	Outcome data reported in review meta-analysis.
Weight loss/decrease	R1	The AEs reported in the trials were only those that occurred in >=20% in either treatment group. Clear outcome was measured but not reported as unlikely to have met the reporting threshold.
Fatigue	NA	Outcome data reported in review meta-analysis.
Somnolence	NA	Outcome data reported in review meta-analysis.
Concentration impairment	R1	The AEs reported in the trials were only those that occurred in >=20% in either treatment group. Clear outcome was measured but not reported as unlikely to have met the reporting threshold.
Speech difficulty	R1	The AEs reported in the trials were only those that occurred in >=20% in either treatment group. Clear outcome was measured but not reported as unlikely to have met the reporting threshold.
Thinking abnormally	NA	Outcome data reported in review meta-analysis.
Ataxia	NA	Outcome data reported in review meta-analysis.

Guberman 2002

**Review outcome**	**Classification**	**Justification for classification**
50% reduction in seizure frequency	NA	Outcome data reported in review meta-analysis.
Seizure freedom	NA	Outcome data reported in review meta-analysis.
Treatment withdrawal	NA	Outcome data reported in review meta-analysis.
Harms data collection method was not recorded.		
Dizziness	NA	Outcome data reported in review meta-analysis.
Headache	R1	The AEs reported in the trials were only those that occurred in >=5% in either treatment group. Clear outcome was measured but not reported as unlikely to have met the reporting threshold.
Nausea/vomiting	R1	The AEs reported in the trials were only those that occurred in >=5% in either treatment group. Clear outcome was measured but not reported as unlikely to have met the reporting threshold.
Paraesthesias	NA	Outcome data reported in review meta-analysis.
Weight loss/decrease	NA	Outcome data reported in review meta-analysis.
Fatigue	NA	Outcome data reported in review meta-analysis.
Somnolence	NA	Outcome data reported in review meta-analysis.
Concentration impairment	NA	Outcome data reported in review meta-analysis.
Speech difficulty	R1	The AEs reported in the trials were only those that occurred in >=5% in either treatment group. Clear outcome was measured but not reported as unlikely to have met the reporting threshold.
Thinking abnormally	R1	The AEs reported in the trials were only those that occurred in >=5% in either treatment group. Clear outcome was measured but not reported as unlikely to have met the reporting threshold.
Ataxia	R1	The AEs reported in the trials were only those that occurred in >=5% in either treatment group. Clear outcome was measured but not reported as unlikely to have met the reporting threshold.

Korean 1999

Review outcome	Classification	Justification for classification
50% reduction in seizure frequency	NA	Outcome data reported in review meta-analysis.
Seizure freedom	NA	Outcome data reported in review meta-analysis.
Treatment withdrawal	NA	Outcome data reported in review meta-analysis.
Harms data were assessed by physicians from the patient diaries.		
Dizziness	NA	Outcome data reported in review meta-analysis.
Headache	NA	Outcome data reported in review meta-analysis.
Nausea/vomiting	NA	Outcome data reported in review meta-analysis.
Paraesthesias	R1	The AEs reported in the trials were only those that occurred in >=5% in either treatment group. Clear outcome was measured but not reported as unlikely to have met the reporting threshold.
Weight loss/decrease	NA	Outcome data reported in review meta-analysis.
Fatigue	R1	The AEs reported in the trials were only those that occurred in >=5% in either treatment group. Clear outcome was measured but not reported as unlikely to have met the reporting threshold.
Somnolence	NA	Outcome data reported in review meta-analysis.
Concentration impairment	R1	The AEs reported in the trials were only those that occurred in >=5% in either treatment group. Clear outcome was measured but not reported as unlikely to have met the reporting threshold.
Speech difficulty	NA	Outcome data reported in review meta-analysis.
Thinking abnormally	R1	The AEs reported in the trials were only those that occurred in >=5% in either treatment group. Clear outcome was measured but not reported as unlikely to have met the reporting threshold.
Ataxia	NA	Outcome data reported in review meta-analysis.

Privitera 1996

**Review outcome**	**Classification**	**Justification for classification**
50% reduction in seizure frequency	NA	Outcome data reported in review meta-analysis.
Seizure freedom	E	*"A 75 to 100% reduction in seizure was not experienced by any patient in the placebo group but was observed in 23% of patients who received topiramate 600mg/day and 13% of patients treated with topiramate 800mg/day or 1000mg/day".* Clearly no seizure free events on placebo but the possibility of some events on treatment. Outcome clearly measured but not reported in full.
Treatment withdrawal	NA	Outcome data reported in review meta-analysis.
Harms data were collected and evaluated at each patient visit.		
Dizziness	NA	Outcome data reported in review meta-analysis.
Headache	NA	Outcome data reported in review meta-analysis.
Nausea/vomiting	R1	The AEs reported in the trials were only those that occurred in >=20% in either treatment group. Clear outcome was measured but not reported as unlikely to have met the reporting threshold.
Paraesthesias	NA	Outcome data reported in review meta-analysis.
Weight loss/decrease	Q	*"Weight loss was present in some patients with anorexia; however, weight was not measured at each visit, and a quantitative assessment of weight change was not available".* Clear outcome measurement not taken routinely for all patients
Fatigue	NA	Outcome data reported in review meta-analysis.
Somnolence	NA	Outcome data reported in review meta-analysis.
Concentration impairment	NA	Outcome data reported in review meta-analysis.
Speech difficulty	R1	The AEs reported in the trials were only those that occurred in >=20% in either treatment group. Clear outcome was measured but not reported as unlikely to have met the reporting threshold.
Thinking abnormally	NA	Outcome data reported in review meta-analysis.
Ataxia	NA	Outcome data reported in review meta-analysis.

Rosenfeld 1996 (this was an abstract only)

**Review outcome**	**Classification**	**Justification for classification**
50% reduction in seizure frequency	NA	Outcome data reported in review meta-analysis.
Seizure freedom	C	*"Twenty-five percent of topiramate patients (placebo 5%) had>=75% reduction in total seizure frequency and 6% (placebo, none) became seizure free."*<br><br>Clear no patients became seizure free in placebo group; percentage data given for treatment group but no reliable numerator/denominator presented.
Treatment withdrawal	NA	Outcome data reported in review meta-analysis.
Harms data were assessed by physicians from the patient diaries.		
Dizziness	NA	Outcome data reported in review meta-analysis.
Headache	R1	Most common harms were listed only (no threshold specified). Clear outcome was measured but not reported as likely to have been uncommon.
Nausea/vomiting	NA	Outcome data reported in review meta-analysis.
Paraesthesias	R1	Most common harms were listed only (no threshold specified). Clear outcome was measured but not reported as likely to have been uncommon.
Weight loss/decrease	R1	Most common harms were listed only (no threshold specified). Clear outcome was measured but not reported as likely to have been uncommon.
Fatigue	NA	Outcome data reported in review meta-analysis.
Somnolence	NA	Outcome data reported in review meta-analysis.
Concentration impairment	R1	Most common harms were listed only (no threshold specified). Clear outcome was measured but not reported as likely to have been uncommon.
Speech difficulty	R1	Most common harms were listed only (no threshold specified). Clear outcome was measured but not reported as likely to have been uncommon.
Thinking abnormally	NA	Outcome data reported in review meta-analysis.
Ataxia	NA	Outcome data reported in review meta-analysis.

Sharief 1996

Review outcome	Classification	Justification for classification
50% reduction in seizure frequency	NA	Outcome data reported in review meta-analysis.
Seizure freedom	NA	Outcome data reported in review meta-analysis.
Treatment withdrawal	NA	Outcome data reported in review meta-analysis.
Harms data were collected by interviewing patients in a non-directed manner at each study visit.		
Dizziness	R1	The AEs reported in the trials were only those that occurred in >=10% in either treatment group. Clear outcome was measured but not reported as unlikely to have met the reporting threshold.
Headache	NA	Outcome data reported in review meta-analysis.
Nausea/vomiting	R1	The AEs reported in the trials were only those that occurred in >=10% in either treatment group. Clear outcome was measured but not reported as unlikely to have met the reporting threshold.
Paraesthesias	R1	The AEs reported in the trials were only those that occurred in >=10% in either treatment group. Clear outcome was measured but not reported as unlikely to have met the reporting threshold.
Weight loss/decrease	NA	Outcome data reported in review meta-analysis.
Fatigue	NA	Outcome data reported in review meta-analysis.
Somnolence	NA	Outcome data reported in review meta-analysis.
Concentration impairment	NA	Outcome data reported in review meta-analysis.
Speech difficulty	NA	Outcome data reported in review meta-analysis.
Thinking abnormally	R1	The AEs reported in the trials were only those that occurred in >=10% in either treatment group. Clear outcome was measured but not reported as unlikely to have met the reporting threshold.
Ataxia	R1	The AEs reported in the trials were only those that occurred in >=10% in either treatment group. Clear outcome was measured but not reported as unlikely to have met the reporting threshold.

Tassinari 1996

**Review outcome**	**Classification**	**Justification for classification**
50% reduction in seizure frequency	NA	Outcome data reported in review meta-analysis.
Seizure freedom	NA	Outcome data reported in review meta-analysis.
Treatment withdrawal	NA	Outcome data reported in review meta-analysis.
Harms data collected by interviewing patients in a non-directed manner.		
Dizziness	NA	Outcome data reported in review meta-analysis.
Headache	NA	Outcome data reported in review meta-analysis.
Nausea/vomiting	NA	Outcome data reported in review meta-analysis.
Paraesthesias	R1	The AEs reported in the trials were only those that occurred in >=10% in either treatment group. Clear outcome was measured but not reported as unlikely to have met the reporting threshold.
Weight loss/decrease	NA	Outcome data reported in review meta-analysis.
Fatigue	NA	Outcome data reported in review meta-analysis.
Somnolence	NA	Outcome data reported in review meta-analysis.
Concentration impairment	NA	Outcome data reported in review meta-analysis.
Speech difficulty	R1	The AEs reported in the trials were only those that occurred in >=10% in either treatment group. Clear outcome was measured but not reported as unlikely to have met the reporting threshold.
Thinking abnormally	NA	Outcome data reported in review meta-analysis.
Ataxia	R1	The AEs reported in the trials were only those that occurred in >=10% in either treatment group. Clear outcome was measured but not reported as unlikely to have met the reporting threshold.

Yen 2000

**Review outcome**	**Classification**	**Justification for classification**
50% reduction in seizure frequency	NA	Outcome data reported in review meta-analysis.
Seizure freedom	E	50% reduction in seizure frequency was reported in this trial and therefore the trial must have measured seizure freedom. As the five studies reporting on seizure freedom reported non-significant results, it is likely that seizure freedom was analysed in this trial but not reported because of a non-significant result, especially as the 50% reduction in seizure frequency result was reported to be significant favouring topirimate.
Treatment withdrawal	NA	Outcome data reported in review meta-analysis.
Harms data were inscribed into a diary or reported to the physician by phone. No questionnaires for AEs were used.		
Dizziness	S1	Dizziness was combined with somnolence in the reporting. Clearly measured for both treatment arms.
Headache	NA	Outcome data reported in review meta-analysis.
Nausea/vomiting	NA	Outcome data reported in review meta-analysis.
Paraesthesias	NA	Outcome data reported in review meta-analysis.
Weight loss/decrease	NA	Outcome data reported in review meta-analysis.
Fatigue	T1	All harms appear to be reported with no reporting restrictions. Likely no events.
Somnolence	S1	Dizziness was combined with somnolence in the reporting. Clearly measured for both treatment arms.
Concentration impairment	T1	All harms appear to be reported with no reporting restrictions . Likely no events.
Speech difficulty	T1	All harms appear to be reported with no reporting restrictions . Likely no events.
Thinking abnormally	T1	All harms appear to be reported with no reporting restrictions . Likely no events.
Ataxia	T1	All harms appear to be reported with no reporting restrictions . Likely no events.

Zhang 2011

Review outcome	Classification	Justification for classification
50% reduction in seizure frequency	NA	Outcome data reported in review meta-analysis.
Seizure freedom	NA	Not reported in review meta-analysis but noted that there were no seizure free events in either treatment group.
Treatment withdrawal	NA	Outcome data reported in review meta-analysis.
Harm was assessed by the attending physician at the end of each 2-week interval. Data collection came from patient-held diaries.		
Dizziness	S1	Dizziness was combined with somnolence in the reporting. Clearly measured for both treatment arms.
Headache	NA	Outcome data reported in review meta-analysis.
Nausea/vomiting	T1	All harms appear to be reported with no reporting restrictions. Likely no events.
Paraesthesias	NA	Outcome data reported in review meta-analysis.
Weight loss/decrease	NA	Outcome data reported in review meta-analysis.
Fatigue	NA	Outcome data reported in review meta-analysis.
Somnolence	T1	All harms appear to be reported with no reporting restrictions . Likely no events.
Concentration impairment	T1	All harms appear to be reported with no reporting restrictions . Likely no events.
Speech difficulty	NA	Outcome data reported in review meta-analysis.
Thinking abnormally	T1	All harms appear to be reported with no reporting restrictions . Likely no events.
Ataxia	T1	All harms appear to be reported with no reporting restrictions . Likely no events.

Coles 1999 (this was an abstract only)

**Review outcome**	**Classification**	**Justification for classification**
50% reduction in seizure frequency	E	"Seizure severity was measured using the Liverpool Scale (LS) and National Hospital Seizure Severity Scale (NHS3). Seizure frequency was recorded throughout by diary".<br><br>Clear outcome measured. Likely analysed from data collected in diary.
Seizure freedom	E	From above - clear outcome measured and likely analysed from data collected in diary.
Treatment withdrawal	G	This outcome was not mentioned in the abstract. However this is an important outcome in this context and was measured and reported in all other studies. Clinical judgement says likely measured.
No data reported on harms.		
Dizziness	S2	No data on harms presented, perhaps due to space limitations (this was reported as an abstract only). Judgment suggests that it is likely that any harm was measured. The decision was based on both clinical judgment and what was reported in all other studies.
Headache	S2	As for dizziness.
Nausea/vomiting	S2	As for dizziness.
Paraesthesias	S2	As for dizziness.
Weight loss/decrease	S2	As for dizziness.
Fatigue	S2	As for dizziness.
Somnolence	S2	As for dizziness.
Concentration impairment	S2	As for dizziness.
Speech difficulty	S2	As for dizziness.
Thinking abnormally	S2	As for dizziness.
Ataxia	S2	As for dizziness.
